# Influence of Manufacturing Technology on the Structure of 80W–20Re Heavy Sinters

**DOI:** 10.3390/ma12233965

**Published:** 2019-11-29

**Authors:** Tomasz Majewski, Tomasz Durejko, Wiesław Urbaniak, Aneta D. Petelska, Magdalena Łazińska, Dariusz Zasada, Ryszard Woźniak

**Affiliations:** 1 Faculty of Mechatronics and Aviation of MUT, Military University of Technology, Kaliskiego 2, 01-489 Warsaw, Poland; ryszard.wozniak@wat.edu.pl; 2 Faculty of New Technologies and Chemistry of MUT, Military University of Technology, Kaliskiego 2, 01-489 Warsaw, Poland; tomasz.durejko@wat.edu.pl (T.D.); magdalena.lazinska@wat.edu.pl (M.Ł.); dariusz.zasada@wat.edu.pl (D.Z.); 3 Faculty of Mechatronics, Kazimierz Wielki University, Chodkiewicza 30, 85-867 Bydgoszcz, Poland; wurban@ukw.edu.pl; 4 Faculty of Chemistry, University of Bialystok, Ciolkowskiego 1K, 15-245 Bialystok, Poland

**Keywords:** W–Re heavy sinters, resistance sintering (RS), pulse plasma sintering (PPS)

## Abstract

Preliminary measurement results of 80W–20Re heavy sinters are presented in this paper. Tested samples were taken from three different technology processes, i.e., resistance sintering (RS), pulse plasma sintering (PPS), and conventional sintering in a vacuum furnace. In the first two cases, the obtained sinters were of similar usable properties (porosity and microhardness), while for vacuum sintering, the material with high porosity was obtained. At the same time, it was found that sintering with the use of electric current (RS, PPS) generates microstructures with highly elongated grains.

## 1. Introduction

Tungsten (W) and tungsten–rhenium (W–Re) alloys are very popular materials used at high temperatures. Recently, some authors have studied the effect of neutron irradiation on the thermal diffusivities of W and W–Re alloys and their temperature dependence before and after irradiation [1], and elucidated the reason for the temperature dependence difference between W and W–Re alloys [2]. Other authors have studied the influence of pre-irradiation thermal treatment and irradiation temperature on the microstructural development of W–26 wt.% Re after neutron irradiation [3].

When an electric current passes through the sinter located in the die, agglomeration of sinter particles occurs, caused by heat energy generated directly in the material [4]. Unlike conventional sintering (resistance furnace), the heat gives off first of all on the particle’s contact area. Due to the specific heating process, methods of this type make it possible to considerably shorten the processes of the production of sintered wares, which often relates to the elimination of some operations present in conventional powder metallurgy, for instance, compaction stage.

**Figure 1:**
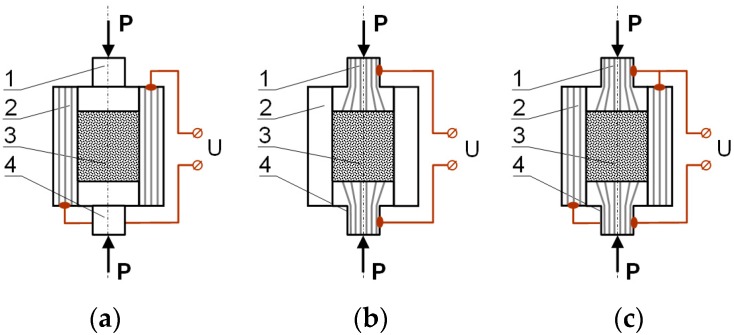
Methods of powder sintering with the use of a passage of electrical current [6]: (**a**) through the material being sintered (a powder), (**b**) through the die, (**c**) simultaneously through the sinter and the die. 1—top electrode, 2—die, 3—sintered powder, 4—bottom electrode.

Among resistance, sintering is numbered processes in which semi-finished or final products are received via the passage of electric current through a sinter or elements of technological equipment (Figure 1). According to this, the following processes can be distinguished [5]:

(a) Sintering caused by the passage of current only through a sinter charge;
(b) Sintering as a result of the passage of current through the die (in this case, mechanisms typical for resistance sintering do not appear);
(c) Sintering in the current flow condition both through the sinter and the die.

The passage of current through the powder generates several phenomena, part of which is characteristic for this type of sintering technique. Heat conditions allow the production of materials of specific mechanical–physical properties that, in turn, induces a high interest in material engineering.

The papers [6,7] consistently showed that resistance sintering significantly accelerates the transportation process of mass between adjacent particles of the powder, which causes its faster consolidation. A distinctive example is the analysis of the results of resistance sinters of chromium [8] and titanium [9], in which it was stated that after several minutes of electric current, the heating densities of the samples were the same as those of comparable samples obtained after a few hours sintering in a furnace, and, moreover, properties of the samples were similar. This comes out of, among others, the high electric field created in the powder/matrix (P/M) compact and a high-temperature gradient appearing at the particle contact area.

Heat exchange with surroundings is an important aspect of the resistance sintering (RS) method. This has a significant influence on the structure and properties of the final material. The highest temperature appears on the sinter axis and decreases towards the sample surface, thus increasing the temperature gradient, due to the worse metallurgic quality that can appear in a superficial (sub-surface) zone, which takes effect in increased porosity and lower hardness. Influence on the metallurgic agglomeration of powder particles in different sinter zones has also die heterogeneity resultant from, for instance, an established way of compaction. Current density distribution in a P/M compact volume can therefore also be non-homogeneous, which leads to substantial temperature differences in different sinter areas [5].

The technologically-advanced variant of sintering with the use of electric current passage through the sintered powder material is the pulse plasma sintering (PPS) process, which has found more and more applications in various fields of the industry. In this case, a high-current electrical discharge of high energy and frequency is used for batch heating. This shortens the duration of the sintering process down to a few minutes [10,11].

The PPS method can be applied to the manufacturing of high-density sinters from a wide variety of materials, i.e., metals (tungsten, titanium, iron) and their alloys, ceramic materials (A1_2_O_3_, TiN, TiB_2_), composites (WC–Co, W–Cu, Cu–diamond, NiAl–TiC, NiAl–Al_2_O_3_), and materials with chemical composition gradient [10,11].

This technique is also recommended for sintering powders of the nano-crystalline structure, since it allows us to receive materials of almost theoretical density with the maintenance of nanometric size of grains. An interesting and far-reaching application of the PPS method is processing using the SHS reaction (self-propagating high-temperature synthesis). Exemplary materials obtained with the SHS method can be sinters based on a matrix of phases from Ni–Al and Fe–Al systems obtained from pure nickel, iron, and aluminum powders [10,11].

In the PPS method, electric current in the form of high energy pulses passes both through the die and the consolidated powder charge. Current strength during condenser bank discharge reaches a value of several tens of kA, and its duration amounts to hundreds of microseconds.

Described in this paper, research relates to heavy sinters of 80W–20Re produced according to different technologies. This material, apart from its very good mechanical properties [12], is characterized by good electrical conductivity, high corrosion resistance, and high resistance to electric arc effect. Tungsten–rhenium alloys have several times greater ability to deform plastic than pure tungsten, due to there being an opportunity to form elements produced from these sinters in the way of fast plastic working, in both hot and ambient temperatures.

For many years investigations have been carried out on the application of these alloys for armor-piercing penetrating cores [12,13], high-current electric contacts, and for the production of some thermoelements, especially for temperature measurements in the range of 1700–2300 °C [14,15].

**Figure 2:**
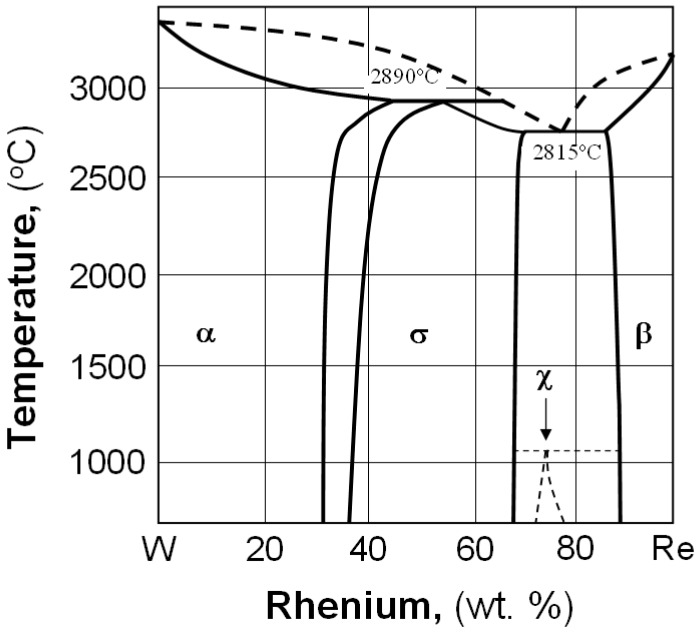
W–Re equilibrium phase diagram.

According to the W–Re equilibrium phase diagram (Figure 2 [16]), there are three solid solutions: α, σ and β, and the metastable phase of χ. Above 26 wt.% of rhenium content in tungsten, a brittle, hard inter-metallic phase is created, and that is why only compositions with low rhenium content are taken into consideration for industrial applications [3].

## 2. Materials and Methods

Heavy sinters 80W–20Re were manufactured using input charge in the form of tungsten powder of 99.99% purity and an average particle size of 2.5 μm, produced by BAILDONIT—g“Węgliki Spiekane” (Katowice, Poland)—and metallic rhenium obtained via the reduction of ammonia VII perrhenate (NH_4_ReO_4_), produced by the Institute of Non-Ferrous Metals in Gliwice.

A mixture consisting of 80% W and 20% Re (wt.%) was made of technically pure elemental powders. A process of low energy milling was carried out in 10 h in the PULVERISETTE planetary mill produced by the FRITSH company (Idar-Oberstein, Germany).

Tested samples were produced in three technological processes:

- vacuum sintering;
- D/C resistance sintering (resistance sintering using direct current);
- sintering with the use of pulse high current discharge (PPS).

In the first variant, a powder charge was consolidated by the cold isostatic pressing (CIP) technique under a pressure of 300 MPa using SO 5-7451-0 laboratory isostatic press produced by NATIONAL-FORGE company (St. Niklaas, Belgium). Received P/M compacts were then preliminarily sintered in a pipe furnace in a dissociated ammonia atmosphere at a temperature of 1150 °C for 60 min. The sintering process was carried out in the 20VP-411/14 HV vacuum furnace manufactured by the SECO-WARWICK company (Swiebodzin, Poland), and the obtained sinters were heat-treated at a temperature of 1650 °C in 120 min.

The second technological variant included uniaxial compaction of the prepared powder mixture under a pressure of 300 MPa, preliminary sintering (the same as in the first variant), and resistance sintering in the die made of Al_2_O_3_ in an argon atmosphere at a temperature of 1650 °C for 5 min under a pressure of 70 MPa. A current of approximately 1300 A was used. In the last stage (resistance sintering), the device developed at the Faculty of Mechatronics and Aviation in the Military University of Technology was used.

The third technological variant included uniaxial compression under a pressure of 300 MPa and vacuum sintering using the innovative method of high current pulse plasma sintering at a temperature of 1700 °C for 5 min under a pressure of 60 MPa in the graphite die. The maximum value of the current approximately 45 kA was applied.

Pulse plasma sintering was performed with the system installed at the Faculty of Materials Engineering at the Warsaw University of Technology.

The sintering methods described in the paper present different conditions for sinter formation. The RS and PPS methods are carried out under pressure. Sintering is a vacuum that has to be preceded by high-pressure pressing. In the PPS method, a lower pressure during sintering should have been used (than in the resistance sintering (RS)) due to the material of the die (graphite). The needed tests were carried out earlier to determine the optimal parameters of the sintering processes. That made it possible to obtain samples with the lowest porosity.

**Figure 3:**
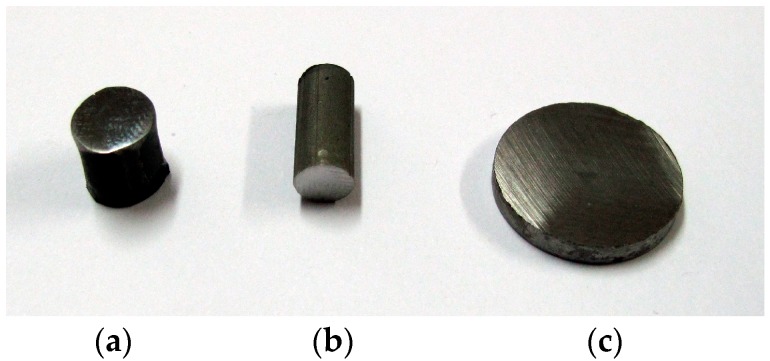
The representative photos of the samples produced with method: (**a**) in the vacuum furnace, (**b**) resistance sintering (RS), (**c**) pulse plasma sintering (PPS).

In Table 1, the sizes and mass of the produced representative samples are presented, and in Figure 3, their representative photos are presented.

Next, the sinters obtained in the above described technological variants were subjected to tests, including chemical and phase composition analyses in selected micro-areas, micro-hardness measurements, and the hydrostatic test of porosity.

An Thermo Fisher Scientific (FEI) Quanta 3D scanning electron microscope (SEM) (Thermo Fisher Scientific, Waltham, MA, USA) with energy dispersive spectroscopy (EDS) was used to determine the chemical composition in micro-regions of the material. The chemical composition was measured using an EDS detector (EDAX AMETEK BV, Tilburg, The Netherlands). An electron backscatter diffraction (EBSD) system coupled with the FEI Quanta 3D field emission gun scanning electron microscope (FEG-SEM) (EDAX AMETEK BV, Tilburg, The Netherlands) was applied for phase analysis of samples. Before the examination, the samples were ground with 400–4000 grit SiC paper, polished with 3–0.25 μm diamond suspensions, and finally polished with 0.1 μm silica suspensions. For analyzing phases, the TSL OIM Analysis 5 commercial software (EDAX AMETEK BV, Tilburg, The Netherlands) was used.

The porosity was determined using the method of hydrostatic weighing in water. The porosity was determined globally for each of the technological variants (three samples per option). Hydrostatic measurement consisted of weighing a dry sample in the air, weighing the sample in water (saturated with water), and after drying, in air.

## 3. Results and Discussion

**Figure 4:**
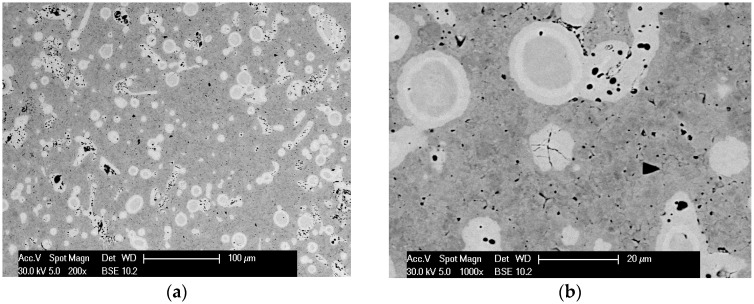
The microstructure of the 80W–20Re sinter produced in the vacuum furnace: (**a**) 200×, (**b**) 1000×.

**Figure 5:**
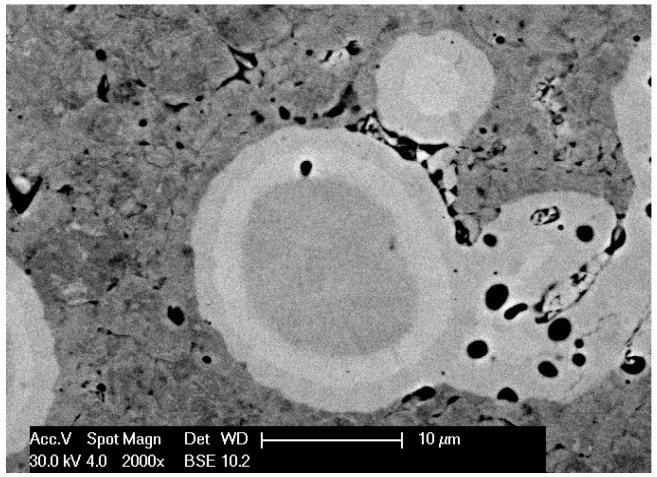
The microstructure of the 80W–20Re sinter in the vacuum furnace: 2000×.

**Figure 6:**
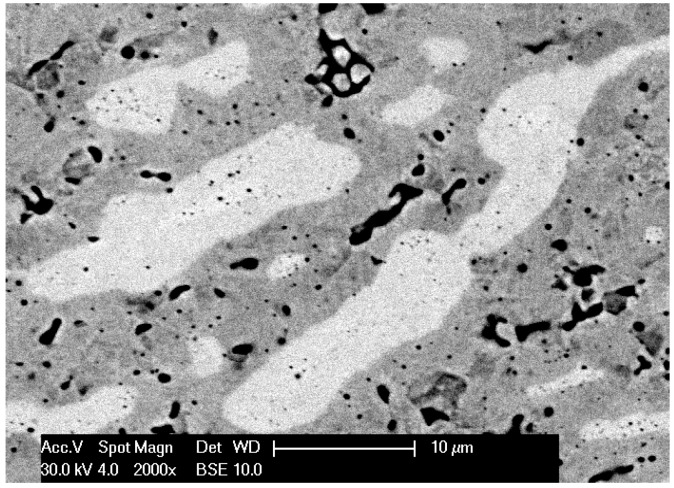
The microstructure of the 80W–20Re sinter produced by the resistance sintering (RS) method (2000×).

**Figure 7:**
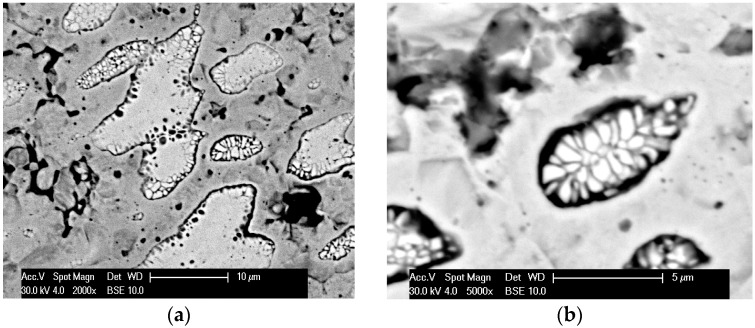
The microstructure of the 80W–20Re sinter produced by the resistance sintering (RS) method: (**a**) 2000×, (**b**) 5000×.

**Figure 8:**
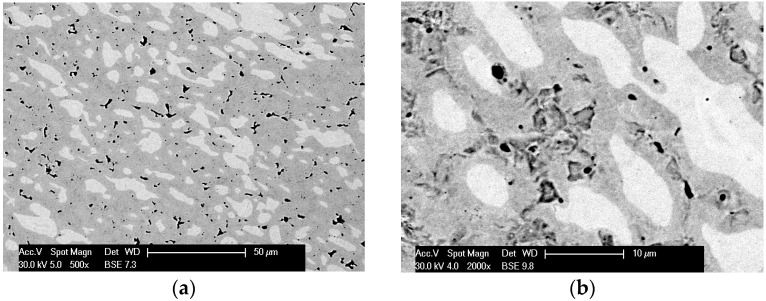
The microstructure of the 80W–20Re sinter produced by the pulse plasma sintering (PPS) method: (**a**) 500×, (**b**) 2000×.

In the first stage of tests, observations of microstructures and determination of phase and chemical composition in selected areas of sinters produced with RS and PPS methods in similar conditions of basis sintering in a vacuum furnace were performed. In Figure 4, Figure 5, Figure 6, Figure 7 and Figure 8, the microstructures of produced 80W–20Re sinters are shown.

In the samples produced using the resistance sintering method, an asymmetry (elongation) of microstructure elements—light grains—was seen. Similar tendencies (however, to a lesser degree) could be observed for samples sintered by the PPS method. For the samples sintered with the RS method, a structure like that seen during solidification (coagulation) was found (Figure 7). One can suppose that during, sintering areas with a temperature higher than melting temperature could exist, and a local melting down of material occurred. For samples produced with the RS and PPS methods, a two-phase structure was observed—light grains surrounded by a dark phase. In some grains, fractures (Figure 4), most likely resulting from a high state of internal stress in the material, were detected.

**Figure 9:**
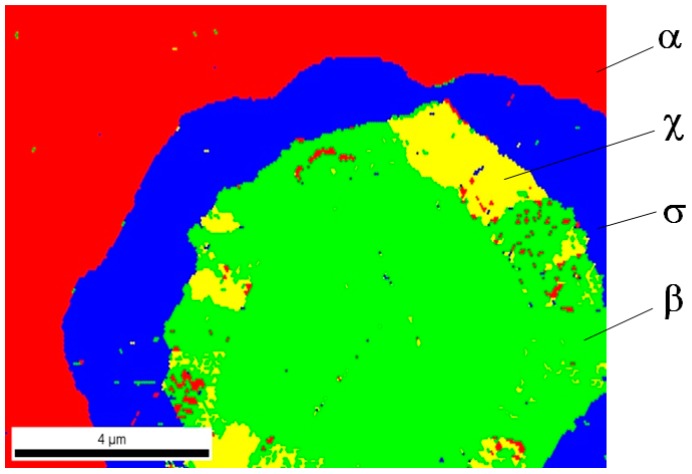
A chart of a phase distribution for the 80W–20Re sinter sample (sintered in the vacuum furnace) in the selected area.

**Figure 10:**
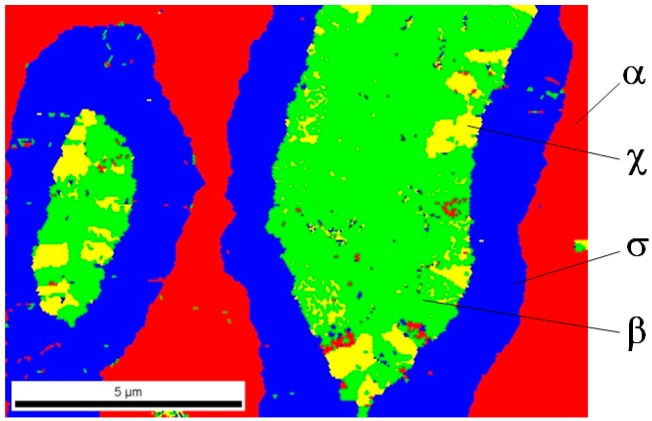
A chart of a phase distribution for the 80W–20Re sinter sample (sintered with the RS method) in the selected area.

**Figure 11:**
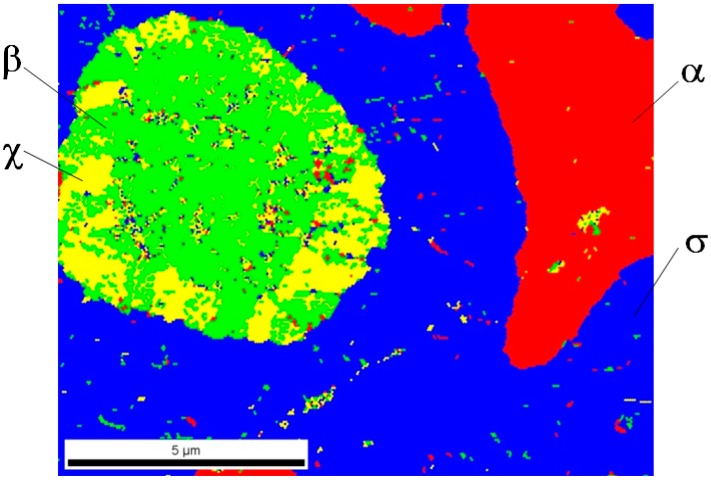
A chart of a phase distribution for the 80W–20Re sinter sample (sintered with the PPS method) in the selected area.

Analysis of phase composition in selected areas of tested samples was carried out using the EBSD method on the Quanta 3D FEG Dual Beam scanning microscope equipped with a system for analysis of backscattered electrons diffraction. In Figure 9, Figure 10 and Figure 11, the identified phases in selected sample areas are presented.

In all cases, it turned out that investigated materials were 4-phase sinters; phase α and 3 successive (direction from outside), phases σ, χ, and β entering into the composition of light grains are marked in red (see Figure 2). Phase distribution in the light grains probably came out of a disruption of diffusion processes of tungsten atoms to rhenium particles. Therefore, the only phase, including first of all rhenium, was revealed inside these objects. One can suppose that a considerable increase in sintering time might drive the structure to be more phase homogeneous, for instance, a two-phase structure.

In Table 2, the EDS measurement results of the chemical composition in selected micro-areas are presented. They were performed using Philips with an EDAX add-on scanning microscope. The presented results show that for samples made with the use of RS and PPS methods, the chemical composition in the individual micro areas was similar. Phase α was composed mainly of tungsten, in which a low amount (~5%) of rhenium was dissolved, whereas in light grains there was an area corresponding to the chemical composition of phase σ (probably due to the finite resolution of the test method).

In the material sintered in a vacuum furnace, a more significant diversity of chemical composition was found. In spherical grains, three characteristic zones could be distinguished—almost pure rhenium (phase β) in the middle, next the rhenium impoverished phase (phase χ, 68.93% Re), and the outside phase, including 59.19% Re (phase σ).

At the next research stage, a porosity of obtained sinters was determined, and micro-hardness measurements in the individual macro-areas of tested samples were made. 

**Figure 12:**
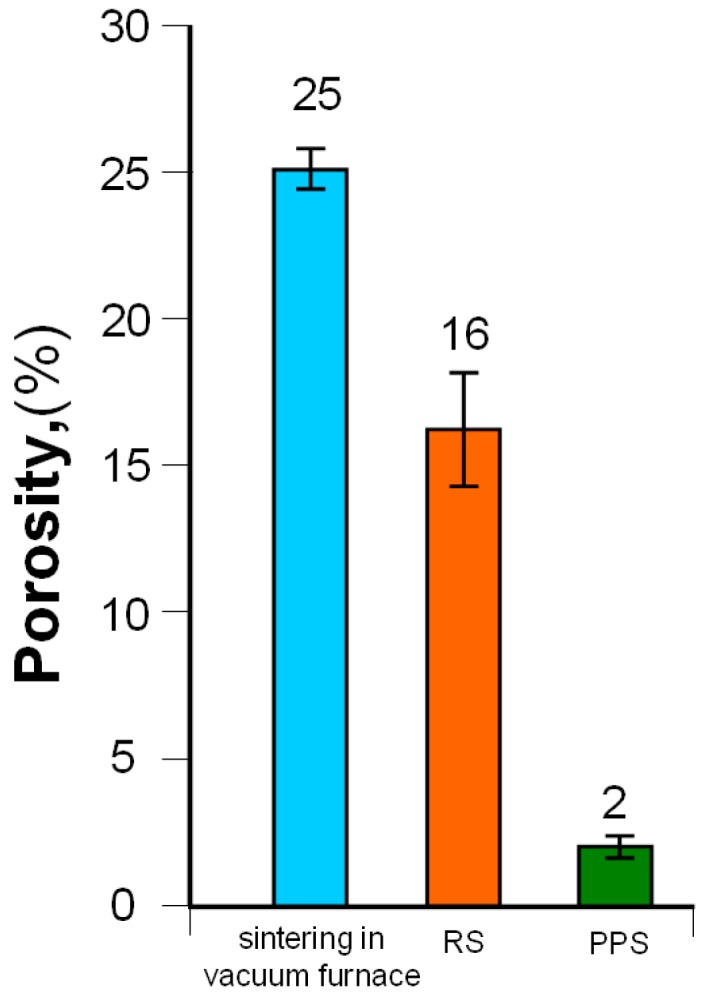
The influence of the applied manufacturing method on the porosity of 80W–20Re sinters.

The PPS sinter had a porosity of 2.0%, which was eight times lower than that for the RS sinter (Figure 12). The sinter obtained by sintering in a vacuum furnace was characterized by the highest porosity (25%).

**Figure 13:**
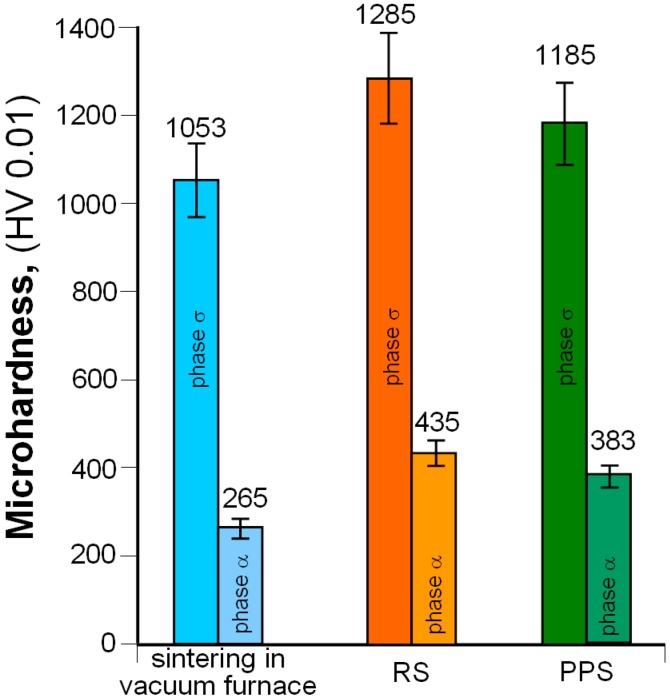
Microhardness of 80W–20Re sinters (for phases α and σ).

Sinter microhardness measurements showed that the highest values of this parameter (for phases α and σ) were found for RS samples (Figure 13). This probably results from the high cooling speed of the material after sintering, which induces high internal stresses in the sintered structure.

**Figure 14:**
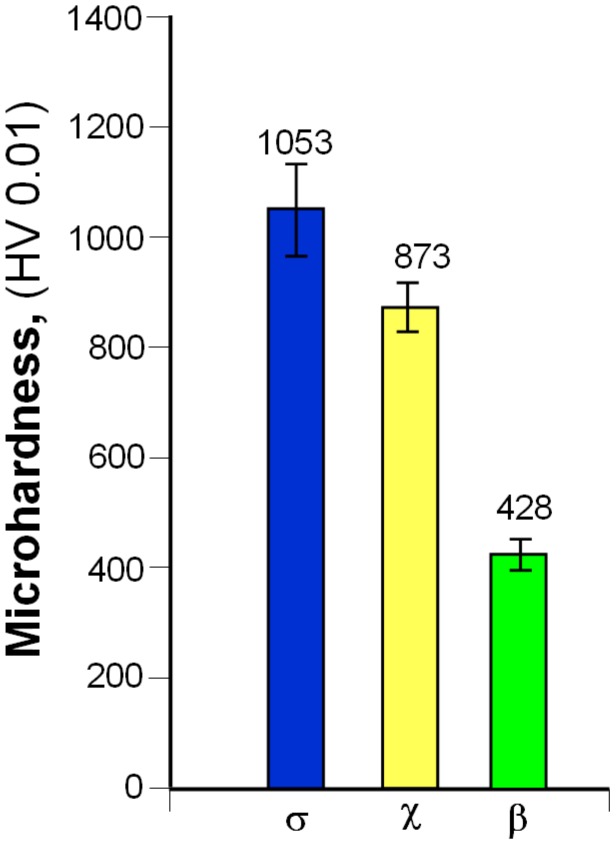
Microhardness of a light phase in 80W–20Re sinters produced in a vacuum furnace.

On the other hand, measurements of microhardness of light grains present in the microstructure of sinters produced in a vacuum furnace (Figure 14) revealed that microhardness decreased with an increase in rhenium content in the investigated phase.

## 4. Conclusions

The presented results are a preliminary stage of research regarding the production of 80W–20Re sinters with different methods. The common conclusion can be drawn that sintering methods using internal heat (RS and PPS) make it possible to obtain material of similar properties, characterized by a higher density as compared to that produced in a vacuum furnace. 

Regardless of the production method, all products were four-phase sinters; therefore, the aim of the ensuing investigations will be an obtainment of a single-phase material that should eliminate the presence of hard and brittle phases. This can be most likely reached by an elongation of the sintering time and an increase in sintering temperature.

Intermetallic phases rising in a sintering process are usually characterized by a high hardness, which results in W–Re sinter brittleness. The following conclusion can be made for the future research: plastic working of these sinters should be performed, maybe hot forging will induce a disruption and uniform dispersion of hard phases—they will compose a strengthening phase.

Therefore, obtained sinters can form semi-finished products from which, after properly projected plastic working (and thermal one), a material of low porosity and good strength properties can be produced.

## Figures and Tables

**Table 1:** The sizes and mass of used samples.

Sintering Method	Size (mm)	Mass (g)
In Vacuum Furnace	Φ8 × 7.5	5.5
RS	Φ6 × 14	6.5
PPS	Φ19.5 × 2.7	15.5

**Table 2:** Elemental contribution (mass percentage) in 80W-20Re sinters.

Sintering Method	Micro Areas of the Light Phase (χ and β Deal with the Sample Sintered in the Vacuum Furnace)							
	Phase σ		Phase χ		Phase β		Phase α	
	W	Re	W	Re	W	Re	W	Re
In Vacuum Furnace	40.81	59.19	31.07	68.93	9.18	90.82	97.61	2.39
RS	40. 36	59.64	-	-	3.45	96.55	93.79	6.21
PPS	41.53	58.47	-	-	2.27	97.73	95.64	4.36
